# Automatic neural mechanisms of social synchrony: pupil and Blink responses in adults with ADHD symptoms

**DOI:** 10.1007/s00702-025-03051-6

**Published:** 2025-10-27

**Authors:** Or Lipschits, Sapir Sadon, Ronny Geva

**Affiliations:** 1 The Department of Psychology, Bar Ilan University, Ramat Gan, Israel; 2 The Susan Gonda Brain Research Center, Bar Ilan University, Ramat Gan, Israel

**Keywords:** Social-Synchrony, ADHD, Eye-Blink, Pupil-Dilation, Automatic-response, Autonomic-reactivity, Simulated-Blink, Dopamine

## Abstract

Human social interactions involve both conscious behaviors, such as speech and gestures, and automatic responses regulated by dopaminergic and noradrenergic neural networks, including pupil dilation and blinking. Dysfunctions in dopamine and noradrenaline activity, common in ADHD, impair automatic social processes such as physiological synchrony and anticipation, leading to broader difficulties in social cognition and interpersonal relationships. Shifting the lens from overt behaviors to automatic processes that lie at the core of social interaction, the present study examined whether adults with ADHD indication (ADHD-Ind) respond differently to a simulated social cue from those without ADHD-Ind. Sixty young adults (16 ADHD-indicated, 44 controls; 20–35 years) watched nine 6-s clips where on-screen eyes blinked twice or four times while eye-tracking recorded blink timing and pupil size. While global measures did not differ between groups, time-locked analyses of blink synchrony to simulated social cues emerged in the control group—particularly around the onset of simulated blinks. In contrast, synchrony effects are attenuated or absent in individuals with ADHD indications. For pupil dynamics, group differences emerged in the sparse simulated blinking condition, where ADHD-Ind showed reduced dilations from − 80 to 560 ms compared to controls and between − 40 and 400 ms compared to the frequent condition. No group differences appeared in the frequent condition. Further, Higher ADHD symptomatology predicted lower blink synchrony and alterations in the pupil time course. The study underscores the added value of automatic, non-verbal synchrony as potential communicative biomarkers for Neuro-Developmental Disorders, and specially in ADHD. Together, the findings suggest that, in alignment with social cue dynamics, timing- and context-specific alterations—rather than global differences—impede social functioning in individuals with ADHD.

## Introduction

Human interaction comprises a rich set of planned behaviors, such as verbal exchange and hand gestures (Goldin-Meadow 1999), alongside spontaneous behaviors and responses, such as autonomic responses (Adolphs 2001) and behavioral mimicry and synchrony (Lipschits and Geva 2024; Prochazkova and Kret 2017). While the planned subset of responses helps us relay our intentions and explain complex notions and ideas (Goldin-Meadow and Alibali 2013), the latter type of responses is perceivable subconsciously (Baron-Cohen et al. 2001), adding an extra layer of meaning (Lipschits and Geva 2024), facilitating emotional connectedness (Hoehl et al. 2021), empathy (Prochazkova and Kret 2017), and anticipation (Adolphs 2001). Some of these autonomic social cues, namely blinking and pupil dilation responses, are thought to be regulated by dopaminergic (Groman et al. 2014; Jongkees and Colzato 2016; Maffei and Angrilli 2018; Ranjbar-Slamloo and Fazlali 2019) and noradrenergic (Laeng et al. 2012; Prochazkova and Kret 2017) inputs. Emerging research highlights the critical roles of dopamine and noradrenaline in modulating these spontaneous social behaviors (Gvirts Probolovski and Dahan 2021; Prochazkova and Kret 2017).

Noradrenaline is vital in regulating attention and emotions—functions that have both top-down and bottom-up effects on socio-emotional self-regulation (Geva and Feldman 2008). Prochazkova and Kret (2017) highlight the role of noradrenaline in mimicry responses and synchronization of autonomic responses, such as heart rate and pupil dilation (Jackson and Sirois 2009; Kret 2015; Mackersie and Calderon-Moultrie 2016). Noradrenaline facilitates the unconscious alignment of physiological states during social interactions, enhancing emotional connectedness, empathy, and mutual understanding (Prochazkova and Kret 2017; Prochazkova et al. 2018). Consequently, it is plausible that disruptions in noradrenergic signaling may impair these subtle yet significant social communication processes, potentially affecting interpersonal relationships and social functioning (Behrens et al. 2019). Dopamine critically influences motor control, reward sensitivity, and attentional processes—functions essential for fluid and responsive social interactions (Arnsten 2009; Groman et al. 2014). Gvirts Probolovski and Dahan (2021) emphasize dopamine’s role in motor planning and interpersonal synchrony, particularly through error detection and adaptive correction during social exchanges. Therefore, atypical dopaminergic signaling may disrupt subtle yet crucial aspects of social interaction, such as timing and synchronization of automatic responses, potentially contributing to broader interpersonal and social challenges (Del Campo et al. 2011; Gvirts Probolovski and Dahan 2021).

Traditionally, Attention Deficit Hyperactivity Disorder (ADHD) symptomatology was associated with dysfunctional prefrontal cortex (PFC) activity, known to be highly dependent on dopaminergic and noradrenergic activity (Bonvicini et al. 2016). When dopamine or noradrenaline levels are too high or too low, the PFC’s regulatory functions diminish, manifesting as inattention, impulsivity, and reduced alertness typical of ADHD (Arnsten 2009). ADHD is a common neurodevelopmental disorder characterized by difficulties with attention, hyperactivity, or a combination of both (American Psychiatric Association, 2013). While the prevalence of ADHD in children is estimated at around 7–10%, it is frequently undiagnosed in adults (Du et al. 2025; Rivas-Vazquez et al. 2023). Individuals with ADHD are known to have social difficulties, including social cognition impairments (Capuozzo et al. 2024; Uekermann et al. 2010), deficits in theory of mind (Bora and Pantelis 2016), and increased sense of loneliness (Jong et al. 2024), pointing to a difficulty in social interactions which accompanies the sense of attention and impulsivity deregulation experienced. Gvirts Probolovski and Dahan (2021) propose a framework for understanding the role of dopamine in motor planning and interpersonal synchrony, which influences error detection and correction during social interactions. According to their framework, atypical dopamine functioning, a characteristic of ADHD (Del Campo et al. 2011), may underpin some of the observed deficits in maintaining interpersonal synchrony, ultimately contributing to broader social challenges (Gvirts Probolovski and Dahan 2021). The noradrenergic system, as well as attention and emotion, are also related to ADHD symptomatology concerning various regulatory functions (Del Campo et al. 2011; Levy 2009).

Given the importance of addressing the gap in the understanding of ADHD in adults and gaps in understanding the social dynamics in people with ADHD, the current study focused on the automatic responses to social cues of individuals who have an ADHD indication compared to those who do not. This choice allows us to test individuals with an ADHD diagnosis, as well as those who are unaware or never diagnosed but perceive themselves as having ADHD symptomatology. We focused on the eyes because the eyes contain a rich array of social information essential to social interaction between people (Baron-Cohen et al. 2001; Schilbach 2015). Gaze location signals the partner’s focus of interest (Carrasco 2011). The degree of pupil dilation indicates the arousal level (Laeng et al. 2012; Zivan et al. 2021), bonding (Lipschits and Geva 2024), and mental effort (Yarmolovsky et al. 2023); and blinking reflects tension and learning (Hoppe et al. 2018; Slagter et al. 2015)—all are important social cues that facilitate understanding and theory-of-mind (Prochazkova and Kret 2017; Prochazkova et al. 2018). Importantly, the eyes are not only a window to the mind of a social partner. They also function as a two-way communication means, sending and receiving signals through automatic mechanisms of mimicry (Prochazkova et al. 2018) that facilitates synchronous interaction with our partner (Prochazkova and Kret 2017).

We explored the automatic responses to a social cue – eye blinks (Hömke et al. 2017; Nakano and Kitazawa 2010; Tatsukawa et al. 2016). We focused on eye blinks because they are perceivable yet, for the most part, unnoticed and automatic, serving a vital role in conveying stress, mental effort, and attention (Holland and Tarlow 1972; Kan et al. 2021). Research has shown that people tend to synchronize their blinking patterns (Nakano and Kitazawa 2010), even with an android, suggesting that blinking pattern synchronizes as a function of social cues. Still, the mechanisms that control blinking remain poorly understood, leaving the question of what neural mechanisms control blinking and their significance in social interaction. Blinking is known to be related to dopaminergic activity, suggesting that an increased eye blink rate is associated with increased dopaminergic activity (Groman et al. 2014; Jongkees and Colzato 2016).

We explored pupil dilation responses in addition to blinking, as both measures are reliable physiological indicators of arousal and noradrenergic activity, each adding its unique input. While spontaneous eye blinks reflect transient fluctuations in dopaminergic and noradrenergic activity associated with attention and arousal states (Demiral et al. 2023, 2024; Jongkees and Colzato 2016); pupil dilation specifically indexes moment-to-moment shifts in autonomic arousal driven primarily by noradrenergic mechanisms (Joshi and Gold 2020). Since observing another person’s eye blinks is a subtle yet salient social cue, we expect this visual input to modulate the observer’s blink pattern and pupil dilation response. Thus, analyzing pupil dilation alongside blinking allows us to capture a broader picture of the underlying arousal mechanisms activated during social interactions involving mutual gaze and blink perception. This combined approach provides a more nuanced understanding of how subtle social signals affect physiological arousal and attentional processes during interpersonal synchrony.

Taken together, we suggest that observing a simulated social cue – eye blink will affect, as previously found, the observer’s eye blink pattern, causing them to synchronize their response to the stimulus. We expect this synchronous blinking to be related to increased arousal and, therefore, to be evident in pupil dilation. Furthermore, we hypothesized that ADHD indication would modulate these relations due to differences in dopaminergic and noradrenergic activity, resulting in variations in eye blink rate and pupil dilation response, as well as a less contingent eye blink response to the stimuli.

Social research demonstrates the significance of imitation and contingent behavioral responses in interpersonal face-to-face communication (Prochazkova and Kret 2017; Schilbach 2015; Shepherd 2010). We explored how neurodevelopmental differences related to ADHD affect eye blink entrainment, with two aims in mind: to deepen the understanding of eye blink significance as a measure of dopaminergic activity and to focus on its communicative value. We focused on internal neurobiological factors manifested in young adults with an indication of having ADHD and without, examining how individual differences related to self-perception as having ADHD-Ind would be expressed in different responsive patterns to blinking entrainment.

Current research shows inconsistent findings regarding blinking patterns in individuals with ADHD (Groen et al. 2017), highlighting the complexity of underlying social processing mechanisms. Investigating these discrepancies through simulations of social stimuli can help clarify how subtle social cues influence blink-related responses in individuals with ADHD symptoms. Furthermore, a differential pupil dilation response might provide additional support and help explain the degree of awareness or effort and the effect the manipulation had on the two groups. We explored eye blink entrainment and pupil dilation response in two ways: once, by computing the average response, exploring the mean effect of manipulation on participants’ blink count and rate, and their mean pupil dilation response, and then, by performing a contingency analysis of eye blink and pupil dilation response evoked before and after each simulated blink.

## Methods

### Participants

The study included a final sample of 60 young adults, 20 to 35, recruited from [removed by AU]. Exclusion criteria ensured that none of the participants had any known genetic or chromosomal syndromes, active psychopathology (other than ADHD), nor reported using psychoactive drugs. Additionally, none of the participants had a head injury history or had any visual impairments or eye conditions aside from glasses. All participants had slept at least 7 h the night before, and did not consume caffeine at least an hour before the experiment. As shown in Table 1, there were no significant group differences in demographic information (age, gender, and residence area). Written informed consent was obtained from all subjects involved in the study. The study was approved by the [Removed by AU] University Psychology Department Ethics Committee (Approval number 2022/10). The research and all methods used followed human research guidelines and regulations.

**Table 1:** Comparison of characteristics between participants screened positive and negative for ADHD

Characteristic	Control (*n* = 44)	ADHD-Ind (*n* = 16)	Test statistic	*p*-value	Effect size
ADHD index	24.21 (7.09)	35.44 (8.05)	*t*(58) = − 5.24	< 0.001	*d* = − 1.53
Age (years)	23.45 (1.88)	23.54 (3.12)	*t*(58) = − 0.14	0.891	*d* = − 0.04
Gender (female)	30	13	χ²(1) = 0.45	0.503	–
Residence type	32 city 6 urban 1 agriculture	11 city 4 urban 0 agri	χ²(2) = 1.23	0.54	–

Note: ADHD index = Adult ADHD Self-Report Scale (ASRS-V1.1) score. Values in parentheses represent standard deviations. Cohen’s d is reported as the effect size for t-tests

### Stimuli

**Fig. 1:**
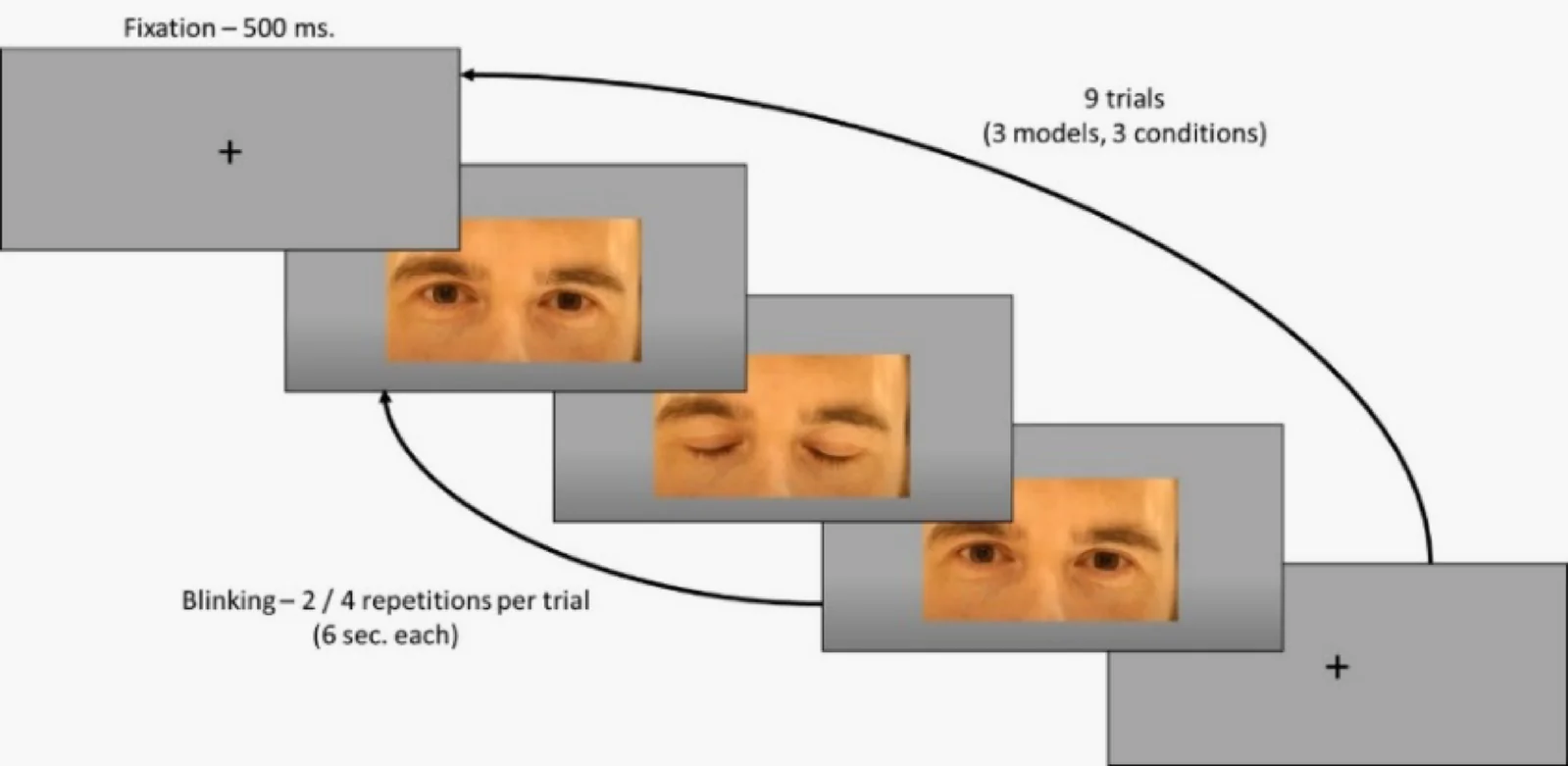
Experimental design. The figure illustrates the timeline of a single trial in the eye blinking experiment. Each trial begins with a fixation cross displayed for 500ms, followed by a video of blinking human eyes under different blinking conditions (2 to 4 repetitions per trial, each lasting 6 s)

Participants sat in front of a computer screen. They were told they were about to watch a set of short videos and that their physiological responses would be recorded. They were told they did not need to do anything, just sit and watch the videos. During the experiment, all participants were shown three sets of videos. The first set showed a change in dynamic pupil size; the second eye blinks, and the third gaze aversion. The current work is based on the data acquired from the blinking set. As seen in Fig. 1, each participant watched 9 videos, 6 s for each video. The stimuli were comprised of 3 models (1 male, 2 female) in 3 conditions – frequent blinking (4 eye blinks), sparse blinking (2 eye blinks), and static (0 eye blinks). Because we observed contingent blinking, we removed the data from the static condition of the analysis. At the beginning of each trial, a fixation cross appeared for 500 ms.

### Data acquisition and processing

Eye tracking data were obtained using a Tobii TX-300 eye tracker (Tobii Technology AB, Stockholm, Sweden), with a sampling rate of 300 Hz, to measure pupil size and gaze coordinates.

Blink detection was done using pupillometry noise according to the method and code described in Hershman et al. (2018), examining both the length of missing eye-tracking data (50–500 ms of missing data) as well as the signal shape of pupil dilation response before and after each segment of missing data to identify eyeblinks. We detected a total of 750 blinks in 60 participants (a mean of 12.295 blinks per participant) across all trials.

Pupil data were processed in RStudio (Team 2021) using the PupillometryR R package (Forbes 2020), according to the guidelines described in Jackson and Sirois (2009). For each trial, right– and left-eye signals were first regression-combined (right on left) and then averaged to a single pupil trace. The series was downsampled to 50 Hz (20 ms bins) using median aggregation, and trials were retained only if they contained ≥ 75% valid samples. We then applied an 11-point median filter and linear interpolation to bridge brief losses due to blinks or tracking noise. Each trial was baseline-corrected by subtracting the mean pupil size during the 500 ms fixation immediately preceding stimulus onset, yielding a baseline-relative pupil signal. We trimmed samples beyond ± 2 SD per subject to limit residual artifacts.

### Procedure

*Eye blink synchrony*. To investigate the degree of synchrony between participants and the simulated eye blink they viewed (between − 500ms to 1250ms relative to simulated blink onset), we followed the method and guidelines of Tatsukawa et al. (2016) and Nakano and Kitazawa (2010). Each time window was divided into 7 equally long bins, and the number of eye blinks that occurred in each bin was counted. We chose 7 bins to allow a time window length that follows previous studies (Tatsukawa et al. 2016) and the time between simulated blinks (minimum 1.5 s). Increased blinking counts in bin pre- or post-simulated eye blinks were interpreted as a measure of contingent response to the simulated blink. We performed a one-sample Z-test to check if the pattern was significant. We compared it to a randomized dataset using the same eye blink rate, a surrogate dataset comprised of randomized eye blinks from the same sample, shuffled 1000 times to create 1000 random samples. For further details on the method used, see Nakano and Kitazawa (2010); Tatsukawa et al. (2016). The current code in R can be found in the supplementry files.

*Pupil dilation response*. We analyzed pupil dilation response around each simulated blink (between − 500ms and 1250ms relative to simulated blink onset). Pupil data were aggregated continuously around each event for each subject individually, creating a mean continuous response for each subject. This method enables us to observe the contingent pupil dilation response to simulated blinks while controlling for the light reflex by analyzing data with minimal luminance change (during video observation only). Differences in pupil dilation changes as a function of time according to simulated blink events were compared by employing consecutive two-tailed t-tests at each time point along the pupil dilation response waveform (Siegle et al. 2008) between participants with or without an ADHD indication and in two conditions (sparse and frequent). To control for high levels of autocorrelation that occur between pupil dilation waveforms, the Guthrie and Buchwald (1991) technique was implemented, using Monte Carlo simulations, to define the region over which continuous points of significant one-tailed t-tests along the waveform could be considered significant (Siegle et al. 2004). Using this technique, our data indicated that a 200 ms window at *p* < .1 is considered significant at *p* < .05.

*ADHD symptomatology* was assessed using the ASRS-V1.1 (Kessler et al. 2005), a 18-item questionnaire rated on a 5-point frequency scale. A sum score across all items was computed and used as a dimensional index of ADHD symptoms, with higher values indicating greater symptom severity (Netzer Turgeman and Pollak 2025). The questionnaire consists of 18 items on a Likert scale (0–4), divided into two parts: (A) for inattentive symptoms and (B) for hyperactivity symptoms. Participants were classified using the WHO ASRS-V1.1 six-item screener (Kessler et al. 2005), where four or more positive responses indicate a likely diagnosis of ADHD. Those who screened negatively for ADHD were classified into the Control group (*n* = 44, M = 24.21, SD = 7.09), and those who screened positively for ADHD were classified into the ADHD-Ind group (*n* = 16, M = 35.44, SD = 8.05). Clinical psychologists analyzed the results to validate the screening. Using a valid screener instead of relying on preexisting ADHD diagnoses ensures standardized measurement across both groups. It provides a valid and reliable criterion for screening, helps detect individuals in the control group who may have undiagnosed ADHD, reduces bias, and enhances the reliability of group classification (Kessler et al. 2005).

## Results

### Average Blink rate, Blink count, and pupil dilation responses as a function of ADHD indication

We first analyzed average measurements to test group (ADHD, Control) differences in blinking patterns and pupil response during each condition (frequent, sparse, static) and their interaction using three Linear mixed-effects models (Table 2). Average measurements were calculated for each participant: mean eye blink count during paradigm; eye blink rate was calculated as the number of blinks per visible minute (valid eye tracking data); and mean pupil dilation response as the mean change of each trial. Six participants were excluded from the analysis: three due to an extreme blink rate (above 2 blinks per second) and three due to missing pupil data (above 75%). Their removal did not alter group differences in ASRS-V1.1 scores, age, gender, or place of residence.

Using global measures, there were no main effects of group or condition, nor were there significant interactions for blink rate. Similarly, no main effects or interactions were observed for mean blink count, and group differences were nonsignificant in all three conditions. For the mean pupil dilation response, the group effect was nonsignificant, and no interactions emerged; however, a main effect of condition indicated a smaller pupil size in the static condition compared to the frequent condition (*p* < .001). ADHD status was not associated with differences in average blink measures or pupil dilation, though pupil size was generally least dilated in the static condition, irrespective of group.

**Table 2:** Comparison of Blink rate, Blink count, and pupil dilation response between participants with and without ADHD-ind across conditions (static, sparse, frequent)

DV	Contrast	Estimate	SE	df	t	*p*
Blink rate (per min)	Frequent: Control – ADHD	5.34	8.97	114	0.6	0.553
	Sparse: Control – ADHD	–0.72	8.93	112	–0.08	0.936
	Static: Control – ADHD	–10.49	8.97	114	–1.17	0.245
Blink count	Frequent: Control – ADHD	0.51	1.37	100	0.37	0.710
	Sparse: Control – ADHD	–0.87	1.37	99	–0.64	0.525
	Static: Control – ADHD	–1.50	1.37	100	–1.10	0.275
Mean pupil dilation	Frequent: Control – ADHD	–0.008	0.023	162	–0.34	0.736
	Sparse: Control – ADHD	0.038	0.023	162	1.62	0.107
	Static: Control – ADHD	–0.037	0.023	162	–1.59	0.114

### The effect of simulated blinking and ADHD indication on contingent blinking response

**Fig. 2:**
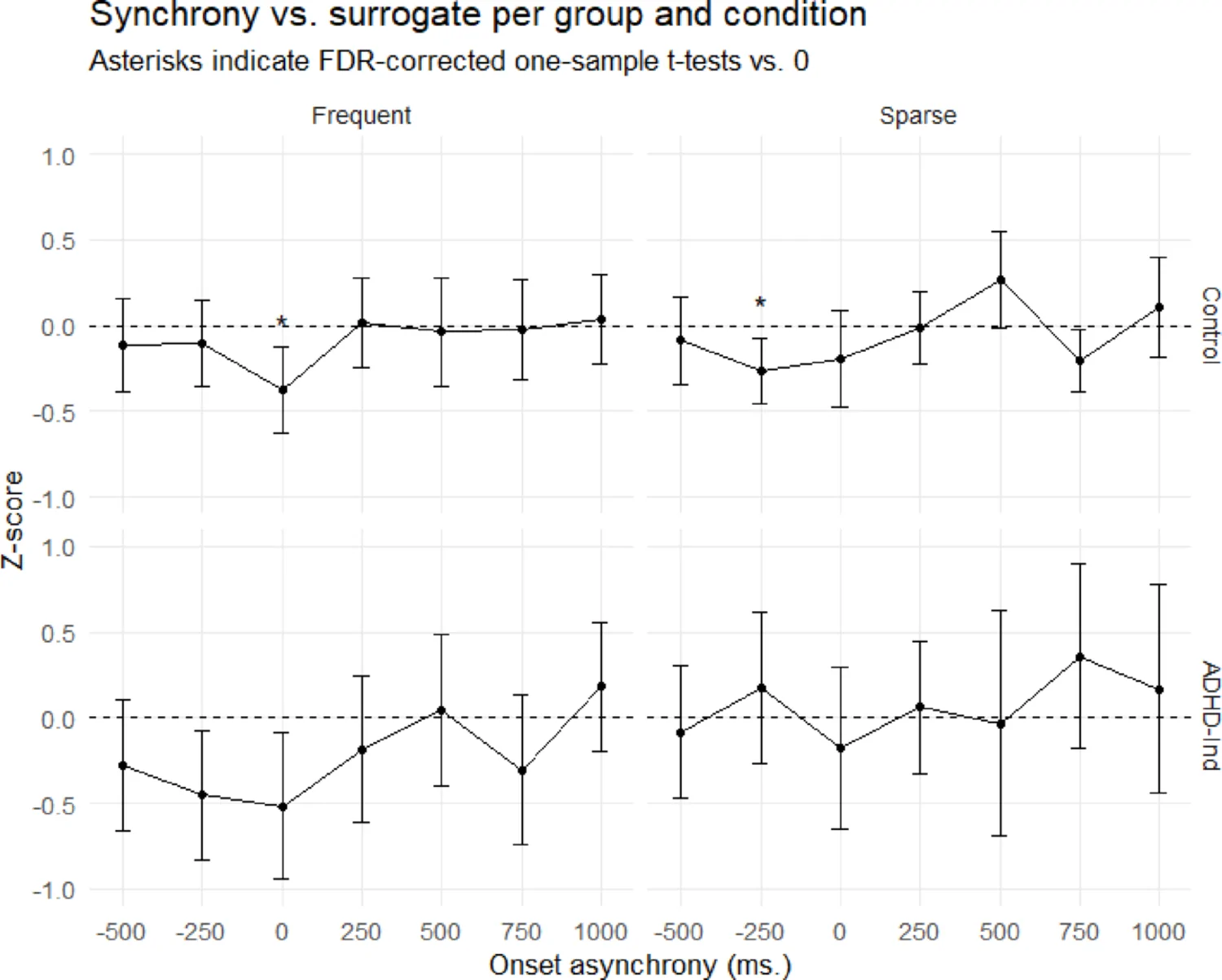
Blink synchrony relative to surrogate data across groups and conditions. Mean Z-scores of blink rate are plotted in 250-ms bins from − 500 to 1000 ms around simulated blink onset (0 ms), separately for the control (top panels) and ADHD-Ind (bottom panels) groups, in frequent (left) and sparse (right) conditions. Error bars represent ± 1 SEM. Asterisks indicate bins where one-sample t-tests against zero were significant after FDR correction. Control participants showed significant reductions in blink rate at 0 ms in the frequent condition and at − 250 ms in the sparse condition, whereas no bins reached significance in the ADHD-Ind group

To examine whether the blinking rate of participants changed in response to simulated blinking appearing in the video, we calculated the mean Z score of blink rate in each 250-ms bin between − 500 and 1250 ms around each simulated blink. A difference in rate occurring in the relevant timing interval of the simulated blink event was referred to as blink synchrony onset. As seen in Fig. 2, participants in the control group displayed evidence of blink synchrony in both the frequent and sparse video conditions. Specifically, one-sample t-tests against zero (FDR-corrected) indicated that in the frequent condition, participants in the control group showed a significant decrease in blink rate at the exact onset of the simulated blink (0 ms), t(42) = − 3.03, *p* = .004, FDR-adjusted *p* = .029. In the sparse condition, control participants also exhibited reduced blink rates just before the simulated blink at − 250 ms, t(42) = − 2.83, *p* = .007, FDR-adjusted *p* = .049, with a trend toward reduced blinking at 750 ms, t(42) = − 2.33, *p* = .025, FDR-adjusted *p* = .086. In contrast, ADHD-Ind participants did not show reliable synchrony effects after FDR correction. Although uncorrected results suggested reduced blink rates around − 250 ms (*p* = .021) and 0 ms (*p* = .22) in the frequent condition, these effects did not survive FDR adjustment (padj = 0.076). No other bins reached significance in the ADHD-Ind group across conditions. Together, these results suggest that blink synchrony to simulated social cues emerges in the control group—particularly around the onset of simulated blinks—. In contrast, synchrony effects are attenuated or absent in individuals with ADHD indications.

### The effect of simulated blinking and ADHD indication on pupil dilation response

**Fig. 3:**
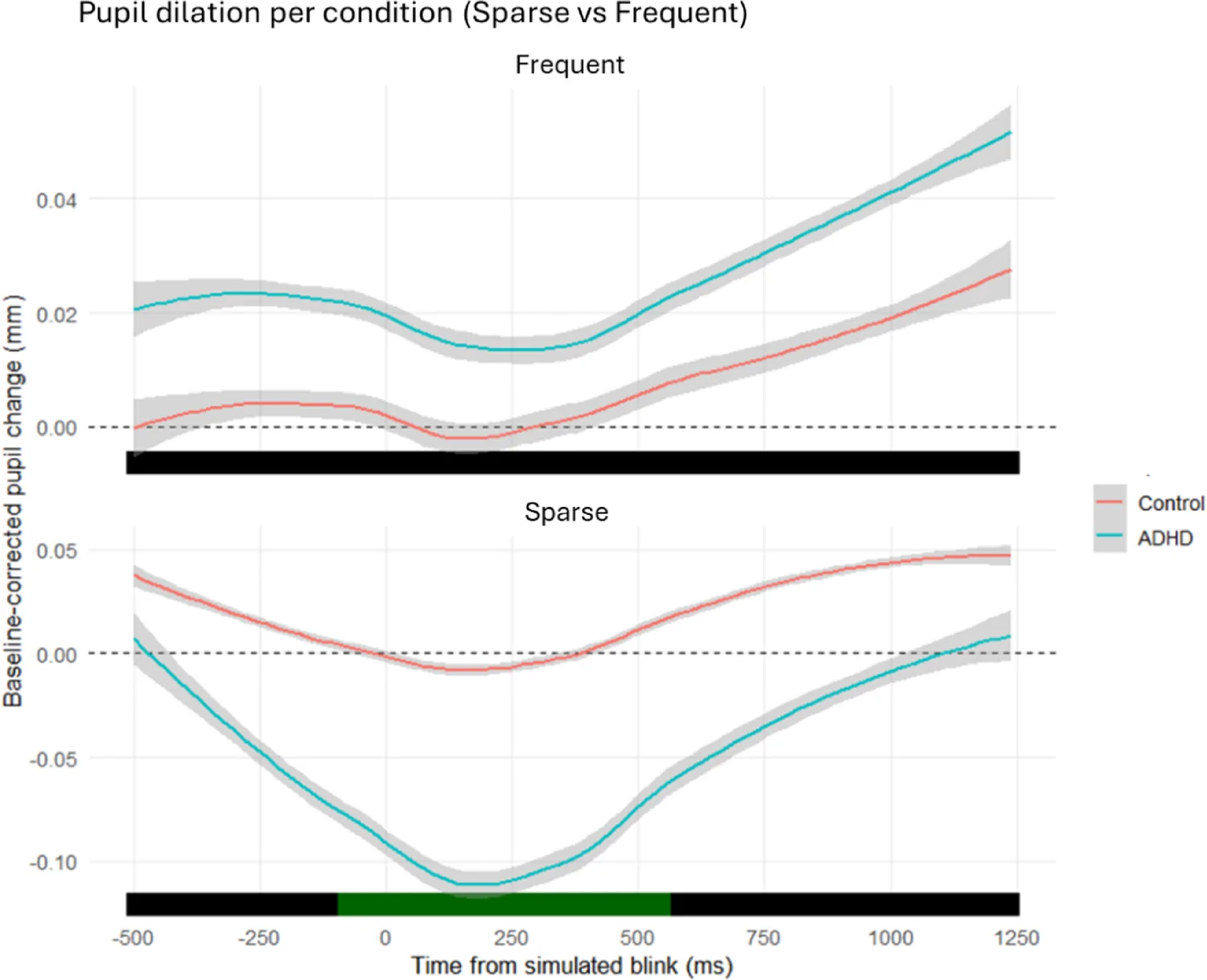
Pupil dilation per condition (sparse vs. frequent). Baseline-corrected pupil change (mm) from − 500 to 1250 ms around simulated-blink onset (0 ms), shown separately for frequent (top) and sparse (bottom) conditions. Lines are group means (Control = red, ADHD-Ind = teal) with shaded 95% CIs; the dashed line marks zero. The bottom rug marks point-wise between-group Welch *t*-tests at each time bin (green = contiguous bins with *p* < .1). No between-group differences appear in the Frequent condition. In contrast, the Sparse condition shows a sustained window of group differences from approximately − 80 to 560 ms (ADHD-Ind more negative/constricted than control)

We analyzed pupil dilation response from − 500 to 1250 ms around each simulated blink to examine the effect of simulated blinks on pupil dilation response. Each simulated blink onset (when the eyes began to close) was used as an event representing time 0. Pupil data was aggregated continuously around each event for each subject individually, creating a mean continuous response for each subject. An independent sample t-test was calculated for each time frame between participants with ADHD-Ind and control in each condition (sparse, frequent). Welch *t*-tests showed no differences in the frequent condition at any time bin. In contrast, the sparse condition exhibited a region of significant between-group differences from − 80 to 560 ms, with ADHD-Ind showing a constricted pupil response compared to control [*t*(36.77) = 2.90, *p* = .006, 95% CI 0.029, 0.162] (Fig. 3).

A one-sample time-course test within groups was performed to examine the different responses to the condition. Results indicated that participants in the control group did not differ from zero at any bin, whereas participants with ADHD-Ind showed a window of significant difference from − 40ms to 400ms.

**Fig. 4:**
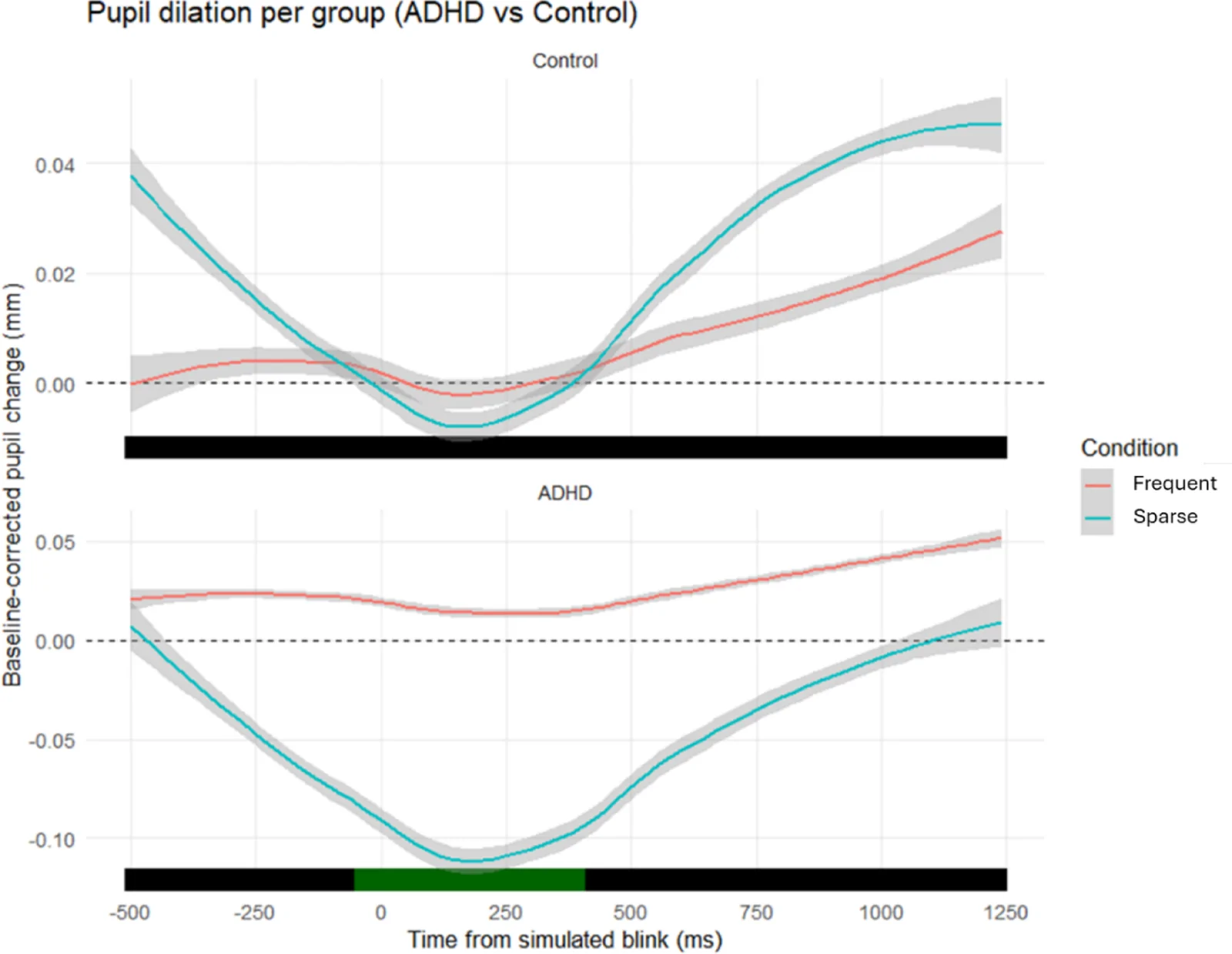
Pupil dilation per group (ADHD-Ind vs. Control). Baseline-corrected pupil change (mm) over the same epoch, faceted by Group (Control top, ADHD-Ind bottom). Lines are condition means (Frequent = red, Sparse = teal) with 95% CIs; the dashed line marks zero. The bottom rug marks point-wise within-group frequent vs. sparse Welch t-tests (green = contiguous bins with *p* < .1). In ADHD-Ind, the sparse response is significantly more negative than frequent over roughly − 40 to 400 ms; no sustained Frequent–Sparse differences are evident in the Control group

Within the ADHD group, the pupil response to sparse blinks was significantly less dilated than its response to frequent blinking when averaged over − 40 to 400 ms [*t*(22.99) = 2.52, *p* = .019, 95% CI 0.022, 0.225], indicating greater pupil dilation around blink presentation to frequent simulated blinks (Fig. 4)—a pattern more similar to that of controls in this condition.

Overall, these results show a condition-specific group difference in participants with ADHD-Ind during sparse blinks, while frequent blinks elicit similar responses across groups.

### The effect of simulated blinking and ADHD symptomatology on contingent blinking response

To better understand the relations between ADHD symptomatology and synchronized blinking response, we analyzed the relations between participants’ synchronized blinking (represented by their Z score), the condition (frequent, sparse), time, and their ASRS-V1.1 total score. For that purpose, we fitted a linear mixed-effects model to standardized blink synchrony.

$$ \:Z\sim \:{\mathrm{ADHD}}\:{\mathrm{symptomatology}} \times \:{\mathrm{Time}} \times \:{\text{Condition + }}\left( {{\mathrm{1}}\left| {{\mathrm{Subject}}} \right.} \right) $$

To explore the triple interaction between ADHD symptom severity, time and blink rate conditions, the number of comparisons tested was reduced to four bins around the simulated blink. Results showed a significant negative main effect for ADHD symptomatology, such that higher ADHD symptom scores predicted lower blink synchrony (* b=-0.224*, * SE=0.094*, $\:t\left(352\right)=-2.37$, * p=.018*, 95% CI [− 0.409, − 0.039]). Neither the time (* b=0.160*, * SE=0.146*, $\:t\left(352\right)=1.09$, * p=.275*), nor the condition (sparse, frequent) (* b=0.102*, * SE=0.133*, $\:t\left(352\right)=0.77$, * p=.444*), reached significance. All interactions were insignificant (ADHD symptomatology × time: * p=.111*; ADHD symptomatology × Condition: * p=.402*; time × Condition: * p=.951*; ADHD symptomatology × time × condition: * p=.482*), indicating the effect of ADHD symptomatology was fairly constant across time and conditions. Semi-partial $\:{R}^{2}$estimates indicated that the fixed effects jointly explained 3.3% of the variance in blink synchrony (95% CI [1.8%, 9.8%]). The unique contribution of ADHD symptomatology was 1.7% (95% CI [0.1%, 5.4%]), a small significant effect.

### The effect of simulated blinking and ADHD symptomatology on contingent pupil dilation response

To examine how ADHD symptomatology relates to the pupil-dilation response, we modeled baseline-corrected pupil change across time and condition using a linear mixed-effects model.

$$ \begin{aligned} \:Pupil\:Dilation\sim \: & {\mathrm{ADHD}}\:{\mathrm{symptomatology}} \times \:({\text{Time + Time}}^{2} ) \\ & \times \:{\text{Condition + }}({\mathrm{1}}{\mid }\:{\mathrm{Subject}}). \\ \end{aligned} $$

To test the triple intecration effect on pupil dilation, we used pupil response from − 500 to 1250 ms around the simulated blink. Results show a change across time after the simulated blink (Time: *b* = 0.0055, *SE* = 0.0010, *t*(≈ 30,000) = 5.68, *p* < .001; Time²: *b* = 0.0048, *SE* = 0.0011, *t*(≈ 30,000) = 4.42, *p* < .001), and a lower pupil dilation response in sparse condition relative to frequent (*b* = − 0.0271, *SE* = 0.0026, *t* = − 10.46, *p* < .001). The main effect of ADHD symptomatology was not significant (*b* = − 0.0269, *SE* = 0.0185, *p* = .151); however, interactions indicated ASRS-dependent differences in trajectory and condition: ADHD symptomatology × Time (*b* = − 0.00283, *SE* = 0.00097, *p* = .0036), ADHD symptomatology × Time² (*b* = 0.00362, *SE* = 0.00109, *p* = .00093), ASRS×Sparse (*b* = 0.02836, *SE* = 0.00257, *p* < .001), Time² × Condition-sparse (*b* = 0.01412, *SE* = 0.00193, *p* < .001), and ADHD symptomatology × Time² × Condition-sparse (*b* = − 0.00432, *SE* = 0.00192, *p* = .025); the Time × Condition-sparse interaction was not significant (*p* = .112). Semi-partial $\:{R}^{2}\:$estimates showed the fixed effects explained 3.0% of variance in pupil dilation, and the unique contribution of ADHD symptomatology was 1.1%, a small significant effect.

Overall, the Results suggest that, unlike global measures of average blink or pupil responses, ADHD-related differences in automatic neural activity emerge in time-locked responses, which are vital in social encounters. The control group showed clear blink synchrony to the social simulated stimuli, whereas the ADHD-Ind group exhibited attenuated synchrony and greater pupil constriction, specifically in the sparse condition. Higher ADHD symptoms were associated with lower synchrony and subtle changes in the pupil dilation time course. Overall, the pattern points to ADHD related timing-specific alterations in alignment to social cues (especially sparse blinks), rather than baseline differences.

## Discussion

The current study provides new insights into the neural networks governing automatic social responses in individuals with ADHD-Ind by examining their behavioral (eye blinks) and physiological (pupil dilation) responses to simulated eye blinks—a subtle yet meaningful social cue. Whereas significant research focuses on overt communicative behaviors such as conversation flow or gesture use, and much of the existing work has emphasized behavioral symptoms, executive function impairments, or structural neural differences, the present study shifts the lens to automatic processes (blinking and pupil dilation, (Hömke et al. 2018; Prochazkova and Kret 2017) offering a window to basic social interaction expressions that may be objectively evaluated. This focus is important because automatic, physiological markers provide an objective and less bias-prone measure of social responsiveness, complementing self-report or observational approaches and offering potential translational value for early identification and intervention.

This perspective also aligns with emerging efforts to identify neurobiological markers of social cognition and arousal regulation. Consistent with calls for integrative models that bridge genetic, biochemical, and behavioral levels of analysis (Bonvicini et al. 2016), the present findings underscore the value of subtle physiological indicators in advancing our understanding of social functioning in ADHD. Specifically, the differential pupil responses we observed across blink conditions (sparse vs. frequent) highlight how stimulus pacing modulates sensitivity to social cues, reinforcing the importance of examining these mechanisms dimensionally.

Given that individuals with ADHD are characterized not only by difficulties in attention and impulsivity but also by impairments in social cognition and interpersonal synchrony (Bora and Pantelis 2016; Capuozzo et al. 2024; Gvirts Probolovski and Dahan 2021). These social challenges in ADHD may stem from time-dependent and trigger intensity-dependent dysregulations in dopaminergic and noradrenergic systems (Arnsten 2009; Del Campo et al. 2011). To date, these were mostly considered to modulate motor planning, emotional regulation, attention, and planned responsiveness to social stimuli (Gvirts Probolovski and Dahan 2021; Joshi and Gold 2020). Here we show that they also affect perceivable automatic social responses, namely, blinking and pupil dilation, which are directly governed by these same neuromodulators (Groman et al. 2014; Jongkees and Colzato 2016). As such, current results offer a unique window into the neurobiological dynamics as well as real-time social reactivity- demonstrating real-time neural transmission effects on social performance.

Our results also highlight the potential of incorporating evaluations of automatic responses to social cues for the research of individual differences. Here, we show that individuals with a higher ADHD symptom load exhibited stronger pupil constriction in the sparse-blink condition, while more frequent blink cues elicited relatively greater pupil dilation, similar to that of controls. These condition-specific effects on blinking and pupil dilation are consistent with the notion that dysregulations in dopaminergic and noradrenergic systems shape both baseline arousal and responsiveness to social cues in time- and intensity-dependent manners.

The automated responses tested, blinking and pupil dilation, not only convey neurobiological information, but also convey communicative value. Blinking is a key component in non-verbal, face-to-face communication (Hömke et al. 2018), serving an essential role in social interaction, conveying to the observing agent moment-to-moment indicators of cognitive understanding, empathy, and stress levels (Hömke et al. 2018; Nakano and Kitazawa 2010; Tatsukawa et al. 2016), or mismatches in these dimensions. Our results support this notion by demonstrating that blink synchrony with social cues is modulated by ADHD symptom severity, indicating difficulties in contingent responding and temporal alignment. The current findings confirm that higher ADHD scores were associated with reduced synchrony to simulated blink cues, particularly around blink onset. Moreover, as the simulated blink frequency decreased (from frequent to sparse conditions), the sensitivity of synchrony measures to ADHD symptoms also increased, indicating a dose–response effect. This pattern suggests that individuals with greater symptom load may struggle with responding appropriately to social cues and align with social partners, due to time and context dependent alternations in reward-based temporal prediction dopaminergic dysfunction (Marx et al. 2022).

Here, we demonstrate that the timing of dopaminergic alterations influences the ability to achieve social synchrony—a crucial social phenomenon that conveys communication and bonding. This was enabled by the experimental method used in this study. The overall higher blink frequency in both groups compared to resting-state baselines (Bentivoglio et al. 1997), which mirrors task-driven increases reported by (Groen et al. 2017), further supports the task’s ecological validity. Critically, the rate does not tell the whole story—analyses of contingent blinking highlight impairments in temporal synchrony with social cues. Individuals with higher ADHD symptom scores showed reduced entrainment to the simulated blink cue, particularly failing to increase blinking after the model blink and instead displaying a relative suppression just before blink onset. Notably, while both groups tended towards momentary suppression of blinking around blink onset, this pattern was present in both sparse and frequent conditions among controls, but only in the frequent condition (trend) among individuals with ADHD. This pattern may reflect altered inhibitory control to avoid missing information (Groen et al. 2017) or a reduced anticipatory capacity, which ultimately results in a missed opportunity for synchrony. Consistent with this, the linear mixed-effects analysis confirmed that higher ADHD symptom severity predicted reduced blink synchrony across conditions, underscoring that impaired alignment is a general feature rather than condition-specific. Given that interpersonal synchrony is a critical foundation of social bonding, communication, and cooperation, its disruption in ADHD may contribute to the broader socio-affective difficulties consistently reported in this population.

These behavioral differences were paralleled by changes in pupil responses. Individuals with higher ADHD symptom load showed stronger pupil constriction in sparse-paced conditions following the simulated blink, suggesting atypical autonomic dynamics consistent with noradrenergic dysregulation (Joshi and Gold 2020; Zhang et al. 2023). This pattern suggests that individuals with ADHD may rely on effortful control mechanisms to remain attentive to social cues. Yet, these compensatory processes appear most challenged under low-arousal (sparse blink) conditions, where the manipulation exerted its strongest impact. This supports the notion that impaired blink synchrony is not solely due to effortful resistance, but also reflects altered sensitivity to subtle social cues, resulting in anticipatory difficulties — emphasizing difficulties in picking up on subtle non-verbal signals (Aduen et al. 2018) and a deficit in signaling the social partner that they are “seen” and are empathized with particularly in less arousing social cues, such as in cases of more sparsed blinking social states ineractions, that typically convey that the partner is fatigued, inattentive, bored, or lacks effort to understand. Importantly, our findings also indicate that as the speed increased, the indicator’s sensitivity decreased, since more frequent blinks are perceived more easily. This finding suggests that stimulus pacing modulates the extent to which blink and pupil responses capture ADHD-related differences. This interpretation is further supported by the significant ASRS × Condition interaction in the linear mixed-effects model, showing that ADHD symptom severity systematically altered pupil dynamics in sparse versus frequent contexts, with the largest differences emerging in the sparse condition.

The integration of blink and pupil data illustrates the intertwined role of dopamine and noradrenaline in social perception and response timing. Our results support a model in which disrupted coordination between these neural systems impairs automatic interpersonal synchrony in a symptom-dependent manner across the ADHD spectrum (Gvirts Probolovski and Dahan 2021; Tripp and Wickens 2009). Moreover, blink synchrony and pupil mimicry foster emotional connectedness (Prochazkova and Kret 2017) and thus play a role in facilitating beneficial relationships (Hoehl et al. 2021). Hence, diminished responsiveness may contribute to a sense of social disconnect and even loneliness, reported among individuals with higher ADHD symptom load (Jong et al. 2024). This interpretation is reinforced by our condition-specific findings: the strongest pupil constriction effects in ADHD emerged in the sparse blink condition, while the mixed-model analysis confirmed that ADHD symptom severity systematically modulated pupil dynamics across conditions. Together, these results highlight that disruptions in synchrony are not uniform but context-dependent, further underscoring their relevance for real-life social interactions.

The findings may shed light on a possible neural and behavioral cascade along the ADHD spectrum: diminished arousal and reward reactivity may reduce the salience of social cues, limiting contingent responses and undermining social rapport. The current approach bridges behavioral and physiological markers, offering an accessible paradigm for screening synchrony-related difficulties and understanding subclinical social dysfunction. Our linear mixed-model analyses further strengthen this interpretation by showing that ADHD symptom severity predicted reduced pupil synchrony across conditions, and that the strongest group differences emerged in the sparse-blink condition. These results point to a systematic, context-dependent attenuation of social cue sensitivity, consistent with the proposed cascade of reduced arousal and impaired anticipation. Finally, from a developmental perspective, the findings may hold implications for early intervention. Synchrony and contingency are foundational in parent-child interaction and predict later functioning in ADHD (Healey et al. 2010). Our work suggests these micro-responses may persist into adulthood, even in undiagnosed individuals, reinforcing the importance of studying individuals with varying levels of ADHD symptomatology.

The study underscores the need to include automatic, non-verbal synchrony markers as valuable communicative biomarkers in Neuro-Developmental Disorders, and specially in ADHD. Pupillary and blinking responses offer promising, non-invasive biomarkers of neural activity in response to social reactivity – integral for personalized approaches to understanding and supporting social communication across the ADHD spectrum. Our findings further show that these markers are sensitive not only to overall ADHD symptom load but also to the frequency of social cues, with the most pronounced differences emerging in sparse-blink contexts. This highlights their potential as fine-grained indicators of social sensitivity, capable of capturing both between-group differences and within-individual variability.

### Limitations

The current study has several limitations. It was conducted on young adults to provide clear insights into the phenomenon in young adults screened positively and negatively for ADHD. Another limitation concerns the gender distribution in our sample, which included more women than men. As the project’s call for participation (and its interest) was on reactivity to social cues, this emphasis may have led to a biased volunteering pattern, with women being more inclined to participate than men.

Previous studies showed no differences in blink rate as a function of age. However, some blinking behaviors and characteristics like velocity (Sun et al. 1997), the effect of task, and gender (Pitigoi et al. 2024) could differ, requiring further research to generalize the results to different age groups. In addition, previous studies highlighted the importance of the eye region during social interaction (Baron-Cohen et al. 2001; Megias-Robles et al. 2020); hence, that was the choice here. Future work may explore the notions of the entire face to explore the effect size limits. Finally, we analyzed the areas of interest and visually checked that the vast majority of gaze behavior data is allocated in the eyes region (see Figure S1. in the supplementary materials). Finally, future work may delve into individual differences in greater depth.

## Data Availability

Data supporting the results of this research will be made available upon request from the corresponding author.
